# Retinology: Green-light ahead for innovation

**Published:** 2018

**Authors:** C. Daniel Brănișteanu

**Affiliations:** * Senior Lecturer, MD, PhD, FEBO

Although Charles L. Schepens, the “father of modern retinal surgery” is credited with establishing retina surgery as a sub-specialty since 1951, the term of retinology nicely defines the *science of the retina*. Actually, ophthalmologists who decide to become highly trained in both diagnosing and treating retinal and vitreous diseases are mainly known as retina specialists and less as retinologists or vitreoretinal doctors.

From scientific work to clinical practice, all retinal fields have marked significant achievements during the last decades. This is easy to understand considering the impressive amount of research and clinical studies performed in both medical and surgical field. 

There is no doubt that, in terms of medical treatment, the intravitreal use of anti-VEGF agents and steroidal implants has marked, from the very beginning, a critical step in the treatment of severe blinding conditions such as vascular occlusions, diabetic retinopathy and neovascular age related macular degeneration (ARMD). Recently, anti-VEGF therapy has become a game changer in diabetic retinopathy, with or without macular edema (DME), due to the approval of this indication and thus the reclaiming of the position of laser photocoagulation, the gold standard of treatment for many decades.

Still, there is a tremendous work underway to evaluate new molecules, both anti-VEGF agents with higher potency and much lower rate of administration or drugs that are more complex, which can enhance the potency of anti-VEGF drugs and reduce the burden of treatment.

There is also a constant need for better treatment protocols and careful monitoring of the clinical study outcomes into real-life settings. Recent data suggest that *Treat-Extend-Stop* regimen might be a better and safer option in patients requiring long-term anti-VEGF intravitreal treatment compared to *Treat and Extend* or *Pro Re Nata* regimens.

Exploring new routes of drug administration for retinal diseases has been a constant concern in the last years. Suprachoroidal delivery of a proprietary suspension formulation of triamcinolone acetonide has recently been associated with significant therapeutic benefit in eyes with naïve or persistent DME with much lower ocular side effects compared to other steroidal delivery methods. At least 2 companies have already announced to date positive preliminary results with topical formulation of anti-VEGF eye drops for neovascular ARMD and DME, but these results need to be confirmed in phase 3 clinical studies. 

A significant progress in the field of ocular gene therapy was obtained last year with the approval for clinical use of an “orphan drug”, voretigene neparvovec (Luxturna™). This adeno-associated virus vector containing human retinal pigment epithelium-specific 65 kDa protein proved consistent and long-term visual improvements were observed in patients with Leber’s congenital amaurosis and retinitis pigmentosa after one subretinal administration. 

The fight against blindness continues even after the approval for clinical use of Argus® II Retinal Prosthesis System, both in United States and Europe, also known as the “bionic eye”. At least 2 systems with different indications are currently under evaluation. PRIMA Bionic Vision System is a miniaturized wireless photovoltaic sub-retinal implant intended to help recovering central visual perception among patients who have lost their central sight due to severe central degeneration or dystrophies. The Orion Cortical Visual Prosthesis System can convert images captured by a video camera into small electrical pulses transmitted wirelessly to electrodes directly implanted on the surface of the visual cortex. This system is basically bypassing both the retina and the optic nerve and virtually has the potential to treat nearly all forms of profound blindness.

Unfortunately, some phase 3 clinical studies failed to confirm the efficacy of promising new therapies for neovascular ARMD (Fovista®), central geographic atrophy (Lampalizumab) or Leber hereditary optic neuropathy (GS010).

In terms of artificial intelligence, robotic surgery can already assist critical steps of vitreo-retinal surgery and medical devices equipped with special software can analyze images and make referral recommendations in diabetic retinopathy and ARMD. 

The non-invasive imaging of the retina was revolutionized by the outstanding development of optical coherence tomography (OCT). Today, OCT technology is present in all retina centers, being an irreplaceable tool for diagnosis, treatment guidance and monitoring. Recently, improvement in acquisition speed rate and software has led to the appearance of OCT angiography. We can now analyze changes in retinal vasculature, at different layers, in a quick, non-invasive, and dye-free manner.

Vitreoretinal surgery continuously evolved regarding efficiency, safety, and instrument compatibility. The shift towards a smaller G (up to 27G) makes vitrectomy quicker and significantly less invasive for the patient. New vitrectomy instruments are more efficient due to the cutting rate of up to 10000 cuts per minute via dual pneumatic drive technology. Due to ultra-high cutting speed capabilities and novel probe geometry, the surgeon can approach the retina much safer and use the probe for different purposes during surgery. Articulated Illuminated Laser Probes, especially adapted for small G surgery, help the surgeon to better view and treat the anterior periphery of the retina. 

The NGENUITY 3D Visualization System, introduced by Alcon in 2016, enhances the surgeon’s comfort, the intraoperative visualization of the retina and is an excellent teaching tool. 

The Vitesse vitrectomy system, exclusively available on the Stellaris Elite system, might also represent a game changer in the future of vitrectomy technology. The ultrasound power (lower than the range for conventional phacoemulsification) is used to generate fluidic and mechanical cutting action for both fragmenting and removing vitreous. This promising new alternative to the currently guillotine-based technology is intended to be much safer, as only the liquefied vitreous near the edge of the port is removed.

During the last decade, retinology has achieved major improvements in imaging, medical, and surgical field with an outstanding dynamic. Thus, the *science of the retina* entered a new era, which is based on better knowledge and more efficient treatment options. There is no doubt that this progression will continue in the search for better and safer treatments.

**Senior Lecturer, Brănișteanu C. Daniel, MD, PhD, FEBO**

